# Risk Factors Associated with HIV Infection among Male Homosexuals and Bisexuals Followed in an Open Cohort Study: Project Horizonte, Brazil (1994-2010)

**DOI:** 10.1371/journal.pone.0109390

**Published:** 2014-10-03

**Authors:** Ana Paula Silva, Marília Greco, Maria Arlene Fausto, Dirceu B. Greco, Mariângela Carneiro

**Affiliations:** 1 Programa de Pós-Graduação em Ciências da Saúde: Infectologia e Medicina Tropical, Faculdade de Medicina, Universidade Federal de Minas Gerais, Belo Horizonte, Brazil; 2 Project Horizonte, Universidade Federal de Minas Gerais, Belo Horizonte, Brazil; 3 Departamento de Alimentos, Escola de Nutrição, Universidade Federal de Ouro Preto, Ouro Preto, Brazil; 4 Departamento de Clínica Médica, Faculdade de Medicina, Universidade Federal de Minas Gerais, Belo Horizonte, Brazil; 5 Departamento de Parasitologia, Instituto de Ciências Biológicas, Universidade Federal de Minas Gerais, Belo Horizonte, Brazil; Rollins School of Public Health, Emory University, United States of America

## Abstract

**Background:**

There has recently been an increase in HIV infection rates among men who have sex with men (MSM). This study aimed at investigating risk factors associated with incident HIV infection in a MSM cohort–Project Horizonte, Belo Horizonte, Minas Gerais, Brazil.

**Methodology:**

This is a nested case-control study in an ongoing open cohort of homosexual and bisexual men, carried out in 1994–2010, during which 1,085 volunteers were enrolled. Each HIV seroconverted volunteer (case) was compared with three randomly selected HIV negative controls, matched by admission date and age (±3 years). During follow-up, 93 volunteers seroconverted and were compared with 279 controls.

**Principal Findings:**

The risk factors associated with HIV seroconversion were: contact with partner’s blood during sexual relations (OR 3.7; 95% CI 1.2–11.6), attendance at gay saunas in search for sexual partners (OR 2.6; 95% CI 1.3–5.4), occasional intake of alcohol when flirting and engaging in sexual activity (OR 2.5; 95% CI 1.3–5.1), inconsistent use of condoms in receptive anal sex (OR 2.4; 95% CI 1.1–5.4), little interest to look up information about AIDS (OR 2.6; 95% CI 1.0–6.7) particularly in newspapers (OR 3.4; 95% CI 1.4–8.1).

**Conclusions:**

This study shows that MSM are still engaging in risk behavior, such as unprotected anal intercourse, despite taking part in a cohort study on various preventive measures. New preventive strategies in touch with the epidemic’s development and the specificities of this particular population are needed.

## Introduction

Data from the Brazilian AIDS/STD Epidemiological Bulletin show that from 1980 to June 2011, 608,230 cases of AIDS were reported in Brazil, out of which 397,662 (65.4%) among male. On average, 35,000 new cases are recorded every year. Between 1990–2010, the main risk of infection among young men (15–24 years) was sexual, mostly in homosexual relationships. There is a significant increase of reported cases among men who have sex with men (MSM), from 25.2% in 1990 to 46.4% in 2010. In 2010, for each 10 heterosexuals living with HIV/AIDS there were 16 homosexuals [1].

There have been constant efforts by the Brazilian Ministry of Health to control the epidemic among this population group. Actions to improve risk awareness and prevention, to confront stigma and discrimination, universal access to HAART and condoms free of charge as well as research funding. Despite these initiatives, the incidence of HIV infection among homosexuals is still high, estimated at 10% in comparison with 0.6% for the general population [2].

Many studies show that MSM are still one of the groups at a higher risk of HIV infection, particularly in countries with concentrated epidemics [3]–[5], and that decreasing infection in this group is a major public health challenge [6].

The reasons for the high rates among MSM include factors linked to sexual practices, namely unprotected anal sex [7]–[9], high number of partners [10], type of partnerships: steady or casual [11] and determinants linked to sociocultural context, such as attendance at male-only saunas and alcohol intake [12]–[15].

It is possible that a unidirectional view of the vulnerabilities of MSM still prevails, one that ignores the particularities of this group in a multifactorial context leading to HIV infection risk practices. Therefore, it is necessary to better understand the factors that hinder the adoption of safe sex practices in order to promote more effective prevention strategies.

This study aimed to investigate risk factors associated with HIV incident infection in a cohort of men who engage in sexual relationships with other men (Project Horizonte/UFMG) in Belo Horizonte, Brazil.

## Methods

### Ethical Statement

The Project Horizonte procedures abide to Brazilian and International Research Ethics guidelines and it has the approval of the Research Ethics committee of the Federal University of Minas Gerais (No. 17750313.0.0000.5149). All participants were required to sign an informed consent form at the Project Horizonte admission.

### Design and study population

This is a nested case-control study in an ongoing open cohort of homosexual and bisexual men (Project Horizonte), carried out to investigate risk factors associated with HIV incident infection.

The Project Horizonte protocol comprises two phases: screening process (recruiting and admission) and follow-up [16]–[17]. Word of mouth (snow ball) has been the main source of recruitment. Various strategies of visibility and attracting volunteers such as pamphlets in bars, nightclubs and GLBT places; partnerships with NGOs, companies; insertions in the mainstream media and team composed of the Project Horizonte volunteers for disclosure to the LGBT community and society in general.

During recruiting, the aim is to identify men who engage in same-sex sexual relations, HIV-negative, over 18 years of age, who live in the Belo Horizonte metropolitan area, and capable of committing to taking part of the planned activities. In this stage, the psychosocial team interviews the volunteer and describes the study plan and objectives. After discussion, the volunteer is offered to sign an informed consent form.

In the follow-up phase the participants are evaluated twice a year to detect incident HIV infection, to investigate risk factors for infection, and to evaluate counseling and educational practices for reducing risk. The visits include psychosocial interview, using a semi-structured questionnaire with 95 questions that includes sociodemographic variables, sexual practices, types of partnership, use of condoms, sexual violence, risk awareness, use of alcohol/drugs, and knowledge and motivation for participating in future HIV vaccine trials. The open questions investigate self-reported sexual identity, the social perception of sexual orientation, and perception of discrimination. During the interview, technical and ethical aspects of anti-HIV vaccine trials are discussed, lab tests are requested (HIV serology, syphilis, hepatitis B and C, and blood tests) and pretest counseling is given. Counseling is an important instrument for breaking the transmission chain of STDs and HIV/AIDS, as it enables reflection on the risks of infection and the need for its prevention. Preventive group interventions are implemented during the follow up period such as discussion forums, workshop on safe sex, and exhibition of films following the discussion. During these activities issues are discussed on sexuality, sexual health, affectionateness, citizenship, STD/AIDS prevention, HIV vaccines, and homoerotic identity. Questions discussed during the forums contributing to individual counseling as do issues brought up in counselling are themes used in the groups.

Those who became infected with HIV during the follow-up are referred to the Infectious and Parasitic Diseases Service (CTR DIP) outpatient clinic where they receive psychological care by the CTR team and medical attendance by the same Project Horizonte health team. To evaluate the infections progression, immunological and virologic evaluations with viral genotyping are performed during follow up until they need antiretroviral therapy, according to Brazilian consensus.

Data from participants who seroconverted during follow-up were compared to HIV-negative volunteers. Cases are volunteers who seroconverted during follow-up in the 1994–2010 period. For each case, three HIV-negative controls were randomly selected, paired by admission date and age (±3 years).

### Variables analyzed

The outcome of interest was HIV incident infection. Seroconversion to HIV was defined by ELISA with confirmatory Western blot tests. Participants who seroconverted during follow-up (cases) were compared to HIV-negative volunteers (controls).

Variables were selected from the psychosocial questionnaire at the time of the seroconversion for cases and at the corresponding visit for controls. Data refers to the six months preceding the interview. The following variables were included:

Sociodemographic variables: Age, education, occupation, self-reported race and monthly income.

Sexual behavior variables: Type of partnership (steady or casual), partner’s sex, type of sexual practice (insertive or receptive anal sex), number of partners, condom use, exchange of sex for money/favors, sexual violence, knowledge about partner’s serology, contact with partner’s blood during sexual practice.

Contextual variables: Contextual variables refer to the relationship of individuals and their sociocultural context and included: Places to look for partners, use of alcohol/drugs, risk awareness, access to information about HIV/AIDS.

Types of partnership were defined as: steady – when subjects reported emotional involvement and continuity of encounters (not necessarily based on partnership duration); and casual – when subjects reported absence of emotional involvement and/or uncertainty of a recurring encounter, including strangers.

Steady and casual partnerships were considered separately, taking into account that subjects could report either kind of partnership, or both.

Condom use was classified as consistent (condoms always used) and inconsistent (condoms not always used). Variations in consistency were related to each kind of sexual practice (receptive anal intercourse and insertive anal intercourse).

### Data analysis

Statistical analyses of the data were performed using STATA version 11.0 software [18]. The HIV infection incidence rate was calculated using the person-years with 95% confidence interval.

Initially, an exploratory analysis of data was carried out by way of summary measures and proportions. Student’s T test was used for means, Kruskal-Wallis’ test for medians, and chi-square for proportions. Analysis was performed using the conditional logistic regression model. Strength of association was determined by odds ratio (OR) at 95% confidence interval. Univariate analysis considered these groups of variables: sociodemographic, sexual behavior, and contextual variables. Variables presenting p≤0.20 in the univariate analysis and variables regarded as relevant for HIV infection in the literature were selected for the multivariate models. Intermediate models were built for each group of variables, and variables with p≤0.10 were selected for the final model. A step-by-step backward selection procedure was used to select the variables in intermediate and final model. Correlation matrix was used to assess the collinearity in each step of the analysis. When collinearity was detected between variables, were chosen the variables that had a better explanatory power. Only adjusted variables showing a significant association (p<0.05) with the occurrence of HIV infection remained in the final model. For definitions of the models, the Hosmer-Lemeshow goodness-of-fit was used [19].

## Results

Between 1994 and 2010, 1,085 volunteers were admitted, and 93 (8.6%) were infected with HIV during follow-up. Two hundred and seventy-nine controls were paired with the 93 identified cases.

Among the 93 seroconversions, 57 (61.3%) occurred up to the fourth follow-up visit, and among those, 20 (21.5%) were already infected in the first follow-up visit six months after admission. The remaining 36 (38.7%) were similarly distributed in the subsequent visits. It is worth noting that 15 seroconversions (20%) occurred among volunteers with over 5 years of follow-up, and two on the 10^th^ year after admission.

The incidence rate for HIV infection in this period was 2.5/100 persons-year (95% CI 2.16–2.9).

### Univariate analysis

#### Sociodemographic characteristics

The sociodemographic characteristics of cases and controls at admission are shown in Table 1. The medians of age at admission were 27 years and at seroconversion 35 years. The comparison of sociodemographic characteristics among cases (93) and matched controls (279) did not reveal any statistically significant difference regarding age, monthly income and occupation. Regarding schooling, cases had a higher proportion of high school and college compared to controls.

**Table 1:** Univariate analysis of the sociodemographic characteristics comparing HIV-positive cases and HIV-negative controls, Project Horizonte, 1994–2010.

	Case	Control	OR(a)	
Variables	N (%)	N (%)	(95% CI)	p-value
Schooling (baseline)				
Elementary school	10 (11.0)	75 (27.2)	1	
High school	62 (68.1)	157 (56.9)	2.96 (1.43–6.12)	0.003
College	19 (20.9)	44 (15.4)	3.29 (1.39–7.76)	0.007
Race				
White	28 (30.9)	90 (31.9)	1	
Pardo	56 (61.5)	136 (49.6)	1.29 (0.77–2.16)	0.328
Black	7 (7.7)	48 (17.5)	0.46 (0.18–1.16)	0.102
Monthly income (BMW)(b)				
<1	14 (15.1)	31 (11.1)	1	1
1–3	50 (53.8)	141 (50.5)	0.79 (0.37–1.68)	0.554
4–6	18 (19.4)	62 (22.2)	0.85 (0.36–1.98)	0.713
>6	11 (11.8)	45 (16.1)	0.40 (0.14–1.13)	0.085
Occupation				
Salaried job	45 (51.1)	162 (61.4)	1	
Self-employed	24 (27.3)	66 (25.0)	1.27(0.58–2.94)	0.229
Unemployed	19 (21.6)	36 (13.6)	1.30 (0.61–2.60)	0.066

(a) OR  =  Matched Odds Ratio.

(b) BMW: Brazilian Minimum Wage.

Data for refusals, “not done” and “don’t know” are not shown.

#### Sexual behavior and use of condoms

Table 2 showed the comparison between cases and controls for sexual behavior and condom use. The majority of participants – both cases (94.6%) and controls (91.4%) – reported engaging in sexual activities only with other men. As to the type of partnership, 64.5% of cases and 67.6% controls reported steady partnerships in the past 6 months. The number of steady partners was not different among cases (mean = 1.2, SD = 0.4) and controls (mean = 1.1, SD = 0.4). Unawareness of the steady partner’s serology was higher among cases (51.7%) when compared to controls (34.0%) (p = 0.011).

**Table 2:** Univariate analysis of sexual behavior, comparing HIV-positive and HIV-negative controls, Project Horizonte, 1994–2010.

Variables	Case	Control	OR(a)	
	N (%)	N (%)	(95% CI)	p-value
Steady partner with men				
No	33 (35.5)	90 (32.4)	1.0	
Yes	60 (64.5)	188 (67.6)	0.87 (0.54–1.42)	0.588
Steady partner with women				
No	92 (98.9)	272 (97.5)	1	
Yes	1 (1.1)	7 (2.5)	2.33 (0.29–18.96)	0.428
Awareness of the steady male partner’s serology				
Partner HIV-negative	24 (40.0)	113 (60.1)	1	
Partner HIV-positive	5 (8.3)	11 (5.9)	4.38 (0.90–21.34)	0.067
Unknown	31 (51.7)	64 (34.0)	2.66 (1.25–5.65)	0.011
Casual partner with men				
No	24 (25.8)	92 (33.0)	1	
Yes	69 (74.2)	167 (67.0)	1.41 (0.84–2.40)	0.193
Casual partner with women				
No	88 (94.6)	266 (95.3)	1	
Yes	5 (5.4)	13 (4.7)	1.17 (0.39–3.42)	0.398
Anal insertive sex				
No	16 (17.2)	61 (22.0)	1	
Yes, condoms alwaysused	31 (33.3)	106 (38.1)	1.40 (0.71–2.72)	0.319
Yes, condoms not alwaysused	46 (49.5)	111 (39.5)	1.66 (0.85–3.26)	0.135
Receptive anal sex				
No	14 (16.0)	73 (26.2)	1	
Yes, condom always used	28 (30.1)	98 (35.1)	1.78 (0.82–3.86)	0.143
Yes, condoms not alwaysused	51 (53.9)	108 (38.7)	2.73 (1.31–5.69)	0.007
Condom rupture				
No	52 (64.2)	180 (78.3)	1	
Yes	29 (35.8)	50 (21.7)	2.38 (1.30–4.35)	0.005
Condom use interferes in sexual intercourse				
No	52 (61.9)	183 (79.9)	1	
Better with condom	4 (4.8)	7 (3.0)	1.97 (0.56–6.91)	0.290
Yes	28 (33.3)	40 (17.4)	2.50 (1.40–4.42)	0.002
Contact with partner’s bloodduring sex				
No	78 (83.9)	263 (94.3)	1	
Yes	10 (10.8)	7 (2.5)	4.19 (1.59–11.0)	0.004
Not sure	4 (4.3)	5 (1.8)	2.64 (0.65–10,76)	0.177

(a) OR  =  Matched Odds Ratio.

Data for refusals, “not done” and “don’t know” are not shown.

Sexual practices were analyzed independently of the type of partnership (steady or casual).

In the past six months casual partnership was reported by 74.2% of cases and 67% of controls. There was no statistically significant difference between number of casual partners among cases (mean = 11.5±15, median = 6) and controls (mean = 9.7±12.8, median = 5), nor in the number of unknown partners (cases mean = 9.2±13.3, median = 4; controls mean = 7.4±11.1, median = 3).

Only one subject (1.1%) among cases and seven (2.7%) among controls reported steady partnerships with women in the past six months and 5 (5.4%) cases and 13 (4.7%) controls reported occasional sex with women.

Cases (82.8%) and controls (78%) reported insertive anal sex in the previous six months, with no significant difference in use of condoms for both groups.

Most cases (84%) and controls (73.8%) reported receptive anal sex in the previous six months. Inconsistent use of condoms was higher among cases (53.9%) than controls (38.7%) (p = 0.007).

Reports that using a condom interfered in the sexual relation were higher among cases (33.3%) than controls (17.4%) (p = 0.002).

Cases mentioned more episodes of contact with partner’s blood during sexual practices (10.8%) than controls (2.5%) (p = 0.004). They also reported higher rates of condom rupture (cases = 35.8%; controls = 21.7%; p = 0.005).

#### Contextual variables

Contextual variables possibly associated to seroconversions were analyzed (Table 3). Most cases (69.9%) and controls (65.1%) looked for sexual partners in sex venues such as nightclubs, gay saunas and public cruising areas. However, attending gay saunas and public cruising areas was more common among cases (53.8% and 39.2% respectively) than controls (20.4% and 16.6%).

**Table 3:** Univariate analysis of the contextual characteristics, comparing HIV-positive and HIV-negative controls, Project Horizonte, 1994–2010.

Variables	Case	Control	OR(a)	p-value
	N (%)	N (%)	(95% CI)	
Attendance at sex venues				
No	28 (30.1)	97 (34.9)	1.0	
Yes	65 (69.9)	181 (65.1)	1.22 (0.75–1.99)	0.417
Attendance at night clubs(b)				
No	22 (33.8)	81 (44.7)	1	
Yes	43 (66.2)	99 (55.3)	1.63 (0.90–2.97)	0.107
Attendance at saunas(b)				
No	30 (46.2)	109 (60.8)	1	
Yes	35 (53.8)	71 (39.2)	1.84 (1.01–3.33)	0.044
Attendance at cruising areas(b)				
No	46 (49.5)	151 (83.4)	1	
Yes	19 (20.4)	30 (16.6)	1.97 (1.04–3.71)	0.037
Alcohol intake during flirting/sex				
No	42 (45.2)	171 (61.7)	1	
Occasionally	44 (47.3)	81 (29.2)	2.18 (1.33–3.80)	0.002
Often	6 (6.5)	15 (5.8)	1.47 (0.53–4.10)	0.460
Always	1 (1.1)	9 (3.3)	0.49 (0.61–3.99)	0.509
Alcohol intake and relation to sex				
No	42 (45.2)	171 (61.5)	1	
Unaltered	17 (18.3)	48 (17.3)	1.41 (0.73–2.75)	0.308
Stimulated	31 (33.3)	55 (20.1)	2.20 (1.28–3.77)	0.004
Inhibited	3 (3.2)	3 (1.1)	4.13 (0.80–21.2)	0.089
Drug use in the last 6 months				
Yes	13 (14.0)	28 (10.1)	1	
No	80 (86.0)	249 (89.9)	1.43 (0.71–2.87)	0.303
Seeking out information aboutAIDS				
No	23 (24.7)	62 (22.3)	1	
Yes	70 (75.3)	215 (77.7)	0.86 (0.49–1.54)	0.621
Seeking out information aboutAIDS in groups/organizationdealing with AIDS				
Yes	4 (4.3)	10 (3.6)	1	
No	66 (70.9)	206 (74.5)	0.83 (0.26–2.76)	0.768
Not seeking	23 (24.8)	62 (21.9)	0.99 (0.28–3.47)	0.990
Seeking out information about AIDS in newspaper				
Yes	9 (9.7)	71 (25.5)	1	
No	61 (65.6)	146 (52.5)	3.74 (1.68–8.35)	0.001
Not seeking	23 (24.7)	61 (219)	3.41 (1.38–8.43)	0.008

(a) OR  =  Matched Odds Ratio (matched by age and admission time).

(b) Only for those that reported attendance of sex venues.

Data for “no applicable” and “no response” are not shown.

There was a significant difference between cases and controls in the occasional intake of alcohol when flirting and/or engaging in sex (cases = 47.3%, controls = 29.2%, p = 0.002). Alcohol was considered an important stimulus for sex among cases than controls (33.3% versus 20.1%; p = 0.004).

Most cases (75.3%) and controls (77.7%) seek out information about AIDS. However, there is little interest in groups or organizations working with AIDS and public testing or counseling services. Cases search for information in newspapers less often than controls (9.7% for cases versus 25.5% for controls), and this was statistically significant.

### Multivariate analysis

The final logistic regression model included variables independently associated with risk of HIV infection, shown on Table 4.

**Table 4:** Final conditional logistic regression model: HIV risk factors among MSM, Project Horizonte, 1994–2010.

Variables	OR(a)	(95% CI)	p-value
Attendance at saunas seeking sexual partners			
No	1		
Yes	2.6	(1.3–5.4)	0.007
Not seeking	1.1	(0.6–2.1)	0.746
Alcohol intake during flirting/sex in theprevious 6 months			
No	1		
Occasionally	2.5	(1.3–5.1)	0.007
Often	1.0	(0.3–3.5)	0.936
Always	0.7	(0.1–6.7)	0.778
Receptive anal sex in previous 6 months			
No	1		
Yes, condoms always used	1.5	(0.6–3.4)	0.381
Yes, condoms not always used	2.4	(1.1–5.4)	0.035
Contact with partner’s blood in previous 6months			
No	1		
Yes	3.7	(1.2–11.6)	0.023
Does not know	3.7	(0.8–16.6)	0.083
Sought information about AIDS in previous 6months in newspapers			
Yes	1		
No	3.4	(1.4–8.1)	0.007
Did not seek	2.6	(1.0–6.7)	0.054

(a) OR  =  Adjusted Odds Ratio (matched by age and admission time).

In the final model remained: contact with partner’s blood during sexual relations (OR 3.7; 95% CI 1.2–11.6), attendance at gay saunas in search for sexual partners (OR 2.6; 95% CI 1.3–5.4), occasional intake of alcohol when flirting and engaging in sexual activity (OR 2.5; 95% CI 1.3–5.1) and inconsistent use of condoms in receptive anal sex (OR 2.4; 95% CI 1.1–5.4). Another statistically significant predictor for seroconversion was little interest to look up information about AIDS (OR 2.6; 95% CI 1.0–6.7) particularly in newspapers (OR 3.4; 95% CI 1.4–8.1).

## Discussion

This study shows that the main factors associated with HIV infection among MSM followed in a cohort study (Project Horizonte) in Belo Horizonte/Minas Gerais are linked to unsafe sexual practices and risk situations in their social context: inconsistent use condom in receptive anal sex, contact partner’s blood during sex, the frequent attendance at saunas to look for sexual partners, and occasional use of alcohol when flirting and/or engaging in sexual relations. Another factor associated with risk was not seeking information about AIDS, particularly in newspapers.

This study explored the relationship between use of condom and type of sexual practice. Those who made inconsistent use of condom (condoms not always used) in receptive anal sex were nearly two times more likely to become infected with HIV. The percentage of seroconversions among those who used condoms inconsistently during this type of practice was 53.9%. A different result was found in a study of MSM in 10 Brazilian cities, for which this percentage was 36.5% [20].

Receptive sex is known to present higher risks than insertive sex [7], [21]–[25]. In evaluating prevention strategies targeted at MSM, Beyrer (2010) pointed out that unprotected receptive anal sex is considered a primary risk for HIV infection and that insertive unprotected anal sex is the primary risk for HIV transmission [23]. Thus, our emphasis is to focus on the discussion of these risks in our interventions.

Use of condoms was reported to interfere with sexual intercourse more often among cases (33%) than controls (17%), as revealed by univariate analysis - enhancing the risk of infection in unprotected receptive anal sex. Another factor to be considered is that minor injuries may occur during unprotected anal sex. In this study, those who reported contact with the partner’s blood during sex were at a 3.8 times higher risk than volunteers who had no such contact.

Other studies show an association between unprotected sexual intercourse with type of partnership: fixed or casual [3], [26]–[31]. In line with these findings, similar cohorts of MSM conducted in São Paulo [17] and Rio de Janeiro [26] (Project Bela Vista and Project Rio) showed a trend for increased risk in receptive anal sex with steady partners. In the Bela Vista cohort unprotected receptive anal sex was reported by 33.8% of those with steady partners (n = 588) and 13.5% of those with casual partners (n = 627). In the Rio project, unprotected receptive anal sex was reported by 33% of those with steady partners (n = 168) and 27% of those with casual partners (n = 180).

Trust and emotional commitment with steady partners can bring a false belief of no risk infection that justifies not using condoms. The issue becomes even more complex when we consider this unprotected practice and ignorance of partner’s serology. Univariate analysis showed that the majority of cases reported not knowing the HIV status of their partners, with a significant number among those with steady partners.

The results indicate that attending saunas and cruising sites to look for sexual partners was more frequent among cases than controls. According to the National Plan to Fight the AIDS and STDs Epidemic among Gays, Men Who Have Sex with Men and Transvestites [2] frequent attendance at non-traditional sociability spaces (e.g. cruising areas, saunas, cinemas, parks, public restrooms) is among the main current contexts of vulnerability. In the present study, those who reported attending such places when searching for partners were 2.6 times more likely to become infected. The possibility of anonymous and impersonal relationships and the high turnover of partners in these spaces configure them as territories for the possible occurrence of unprotected sexual intercourse. These results confirm the data from a sociobehavioral survey conducted over the internet with MSM in the city of Salvador/Brazil [32], which observed that those frequenting saunas and public restrooms tend to engage in unprotected anal sex. Other studies also showed high attendance levels of MSM to these places in search of sex partners [3], [5], [30], [33]–[35].

Use of alcohol was also associated with HIV infection. The well-known effects of alcohol, such as disinhibition and reduced ability to plan actions, may lead to a decreased perception of risk and therefore to forego the use of condoms. A WHO sentinel study [36] conducted among groups of high sexual risk in India indicated that MSM are among the segments with high rates of alcohol consumption (at least once a week) and use of condom is low among gay men, especially when under the influence of alcohol and/or other psychoactive substances, even with knowledge of preventive measures.

A qualitative research at Project Horizonte [17] assessed the representation of risk in different subcategories of sexual orientation self-assessment (gay, bisexual and “*entendido*”, a Brazilian informal term for homosexual). For most volunteers alcohol use, seen as a facilitator of sex, was associated with lack or diminished prevention. These results corroborate other studies [13], [37]–[38] that showed that when alcohol is ingested before or during sex and its use is anchored by the belief that consumption would increase sexual pleasure, it can promote unsafe sexual practices. In a cohort study of MSM conducted in Rio de Janeiro it was observed that 51.5% of individuals followed reported alcohol use before and during encounters followed by intercourse, but only 9.8% reported having engaged in unsafe sex practices [26].

The ways information about AIDS and other STDs are researched and elaborated upon by volunteers in the cohort reflect the value placed on knowledge and its practical application in everyday sex. The findings in this study shows that cases access information about HIV/AIDS and other STDs less frequently than seronegative volunteers, especially in newspapers, and this difference was statistically significant.

Among the limitations of the study, recruitment for the cohort was conducted primarily through snowball sampling, which tends to form a group with homogeneous characteristics. This is reflected in the limited reach to other high-risk subpopulations of MSM (e.g. sex workers and transvestites). As a result, it is possible that the infection rates observed in the Project Horizonte underestimate that of the general population of men who have sex with men. Information bias related to the questions of condom use, which could result in an overestimation of its use, may have been minimized. First, the study’s longitudinal characteristic, facilitates the establishment of a confidence bond between volunteers and Project Horizonte team. Second, volunteers may have felt more comfortable in reporting risk situations or difficulty in condom use with the guarantee of confidentiality and a non-judgmental stance on the reported situations. As another safeguard, the psychosocial questionnaire was designed to detect - at different times of its application - contradictions in the volunteers responses on condom use through questions that address sexual risks in different situations.

Factors linked to sexuality, because of its complexity, are often difficult to measure. Understanding vulnerability goes beyond the scope of objective measures, because involve individual perceptions of risk and its relation to the social context in which sexuality is experienced. The subjective constructs and values involved in the sex scene may not have been fully reached through the quantitative assessment tool used here. Our results show that MSM are still engaging in risk behavior despite taking part in a cohort study with several preventive initiatives.

It is possible that inconsistent use of condom is linked not only individual and social determinants that facilitate exposure to unsafe sexual practices, but also, and especially for the last decade or so to optimistic perceptions that AIDS is no longer characterized as a problem given the effective treatment options, which may have contributed to the reduced visibility of the epidemic in the media.

Thus, current prevention initiatives aimed at a gay and bisexual public focus almost exclusively on the use of condoms for safe sex.

There is a shift in terms of prevention, from the normative aspect of using condoms to preventive alternatives that have as its substrate greater individual autonomy in the choice of protection, and are often different from standard recommendations. The literature shows that MSM seek to reduce risk of infection by employing strategies such as serosorting (choice of sexual partner with the same HIV status), strategic positioning (preference for lower risk anal sex, in which the seronegative MSM opts for insertive sex with partners known to be HIV positive or of unknown serology and receptive sex with partners known to be HIV-negative) and also withdrawal before ejaculation [39]–[42].

In this perspective, this study points to the need to broaden the discussion to include these changes in the field of prevention, considering the specificities of this segment. We must better understand the combined use of different prevention strategies, their effectiveness and impact in combating the epidemic. These approaches, however, must be explored taking into account the importance of condom use.

## Supporting Information

Checklist S1 **STROBE checklist.**
(DOCX)
